# Dysregulated T helper type 1 (Th1) and Th17 responses in elderly hospitalised patients with infection and sepsis

**DOI:** 10.1371/journal.pone.0224276

**Published:** 2019-10-28

**Authors:** John D. Coakley, Eamon P. Breen, Ana Moreno-Olivera, Alhanouf I. Al-Harbi, Ashanty M. Melo, Brian O’Connell, Ross McManus, Derek G. Doherty, Thomas Ryan

**Affiliations:** 1 Department of Intensive Care Medicine, St James’s Hospital, Dublin, Ireland; 2 Trinity Translational Medicine Institute, St James’s Hospital, Dublin, Ireland; 3 Department of Immunology, Trinity Translational Medicine Institute, Dublin, Ireland; 4 Department of Clinical Microbiology, St James’s Hospital, Dublin, Ireland; 5 Department of Clinical Medicine and Genetics, Trinity Translational Medicine Institute, Dublin, Ireland; Universite Paris-Sud, FRANCE

## Abstract

**Objective:**

The role of Th1 and Th17 lymphocyte responses in human infection and sepsis of elderly patients has yet to be clarified.

**Design:**

A prospective observational study of patients with sepsis, infection only and healthy controls.

**Setting:**

The acute medical wards and intensive care units in a 1000 bed university hospital.

**Patients:**

32 patients with sepsis, 20 patients with infection, and 20 healthy controls. Patients and controls were older than 65 years of age. Patients with recognised underlying immune compromise were excluded.

**Methods:**

Phenotype, differentiation status and cytokine production by T lymphocytes were determined by flow cytometry.

**Measurements:**

The differentiation states of circulating CD3^+^, CD4^+^, and CD8^+^ T cells were characterised as naive (CD45RA^+^, CD197^+^), central memory (CD45RA^-^, CD197^+^), effector memory (CD45RA^-^, CD197^-^), or terminally differentated (CD45RA^+^, CD197^-^). Expression of IL-12 and IL-23 receptors, and the transcription factors T-bet and RORγt, was analysed in circulating T lymphocytes. Expression of interferon- γ and IL-17A were analysed following stimulation *in vitro*.

**Results:**

CD4^+^ T cells from patients with infection predominantly expressed effector-memory or terminally differentiated phenotypes but CD4^+^ T cells from patients with severe sepsis predominantly expressed naive phenotypes (p<0.0001). CD4^+^ T cells expressing IL-23 receptor were lower in patients with sepsis compared to patients with infection alone (p = 0.007). RORγt expression by CD4^+^ T cells was less frequent in patients with sepsis (p<0.001), whereas T-bet expressing CD8^+^ T cells that do not express RORγt was lower in the sepsis patients.

HLA-DR expression by monocytes was lower in patients with sepsis. In septic patients fewer monocytes expressed IL-23.

**Conclusion:**

Persistent failure of T cell activation was observed in patients with sepsis. Sepsis was associated with attenuated CD8^+^Th1 and CD4^+^Th17 based lymphocyte response.

## Introduction

Sepsis is a common disease worldwide, accounting for more fatalities than many common cancers[1], however elderly patients experience particularly high mortality rates[2]. Sepsis has been regarded as the clinical manifestation of a cytokine storm. However the animal models supporting this hypothesis do not accurately reflect human sepsis pathophysiology[3]. Recently the role of immunosuppression in the pathophysiology of sepsis in patients has been highlighted[4]. Septic patients exhibit a failure of both innate and adaptive immunity[5, 6] with increased apoptosis, impaired pathogen killing and decreased production of proinflammatory cytokines by a range of lymphocytes [7–9] [10–12]. Decreased antigen presentation by antigen presenting cells is associated with expression of inhibitory receptors on T cells and expansion of T regulatory (Treg) cells [9, 13, 14].

Adaptive immune responses against microbial infections are controlled by CD4^+^ T cells, which differentiate into distinct types of effector T cells. Th1 cells are induced by IL-12 and are characterised by the expression of the transcription factor T-bet and the cytokines interferon-γ (IFN-γ) and tumour necrosis factor-α (TNF-α), promoting macrophage and cytotoxic T cell activation and immunity against viruses and intracellular bacteria. Th17 cells are induced by IL-1β, IL-6, transforming growth factor-β (TGF-β) and express the transcription factor RORγt and release IL-17A, IL-17F and IL-22. These cytokines promote immunity against extracellular bacteria and fungi by inducing neutrophil recruitment, antimicrobial peptide release, and the maintainence of intestinal barrier function [15, 16]. CD8^+^ T cells also show distinct phenotypes with some producing IFN-γ and others producing IL-17.

Previous studies have demonstrated that patients with sepsis show skewed CD4^+^ T cells responses, polarised towards Th2 and Treg responses with depletion and impairment of Th1 cells [13]. In our study, we used in vitro stimulation and flow cytometry to examine Th1 and Th17 pathway activation in circulating naive, central memory, effector memory and terminally differentiated CD4^+^ and CD8^+^ T cells from sepsis patients and control subjects. By quantifying IL-12 and IL-23 production by monocytes and IL-12 and IL-23 receptors, T-bet and RORγt, and IFN-γ and IL-17A expression by CD4^+^ and CD8^+^ T cells, we identified defects in both Th1 and Th17 responses in septic patients. Furthermore, to discern sepsis specific molecular mechanisms from those associated with the occurrence of infection, we included an additional patient group with infection but without sepsis. Given adverse outcome in elderly patients with sepsis, and the occurrence of immune senescence in the elderly [17], this study was performed in elderly hospitalised patients.

## Methods and materials

### Study population

The St. James’s hospital Research and Ethics Committee granted ethical approval for this study. Patients gave written informed consent, or when not possible, the next of kin assented to the study as per guidelines of National Consent Advisory group[18]. Blood samples, demographic data and clinically relevant information were collected from 42 patients in a septic group (32 for immune-phenotyping, and 10 for cell stimulation), 30 patients for the infection group (20 for immune-phenotyping, and 10 for cell stimulation), and 30 patients for the control group (20 for immune-phenotyping and 10 for cell stimulation). The study was performed from 2016 to 2018. Patients admitted to the intensive care unit (ICU) in St James’s Hospital with septic shock, according to the Sepsis 3 definitions, were screened for inclusion in the study. Blood samples were taken within 72 hours of admission and weekly thereafter for a further 3 samples until death or discharge from hospital. Patients with infection were recruited from hospital wards in collaboration with the clinical microbiology service. In the infection group blood samples were taken within 72 hours of positive culture and a second blood sample was taken a week later unless the patient was discharged from hospital. One patient in the infection group was removed due to a very high bilirubin causing the flow cytometry to be uninterpretable, and so reducing the patient number to 19. The healthy control group was recruited from the community; these subjects were attending a hospital phlebotomy service for community-based family practice and did not have any current or recent infections. Patient demographics are shown in Table 1.

**Table 1:** Demographic and Clinical Data.

Clinical Data		Immunophenotype Study			Stimulation Experiment		
		Control	Infection	Septic	Control	Infection	Septic
**n**		20	19	32	10	10	10
**Age**		73 [69–78.25]	81.5 [70.25–87.25]	73.5 [68.75–79.25]	72 [67–74.5]	85 [68.75–87.5]	57.5 [53.75–69.25]
**Male Gender**		6 (30%)	11 (58%)	20 (62.5%)	5 (50%)	6 (60%)	4 (40%)
**APACHE score**		N/A	12.5 [8–16.5]	19 [16–24.5] p<0.0001	N/A	14 [10–14]	21.5 [16.25–24.5]
**SAPS score**		N/A	N/A	48 [37.75–54.5]	N/A	N/A	49 [38.25–55.75]
**SOFA score** **on admission**		N/A	3 [1.75–4]	7 [5.75–10] p<0.0001	N/A	2 [0–3]	10 [7.5–11.75]
**SOFA score on day of first sample**		N/A	1 [0.75–1.25]	7 [5–8.25]	N/A	1 [0–1]	8 [4.5–9.75]
**Time to 1**^**st**^ **sample from admission (days)**		N/A	2.5 [2–3]	1.5 [0.75–2]	N/A	3 [2.5–4.5]	5 [4–6]
**ICU duration (days)**		N/A	N/A	14.5 [8.75–33.25]	N/A	N/A	17.5 [9.25–26]
**Mortality in ICU**		N/A	N/A	11 (34.4%)	N/A	N/A	1 (10%)
**Mortality in Hospital**		N/A	0	13 (40.6%)	N/A	1 (10%)	3 (30%)
**Inotropic Support**		N/A	0	30 (93.75%)	N/A	0	10 (100%)
**Days on inotropes**		N/A	0	7 [3–13]	N/A	0	7.5 [6–10.5]
**Invasive ventilation**		N/A	N/A	28 (87.5%)	N/A	N/A	9 (90%)
**Days on invasive ventilation**		N/A	N/A	14.5 [5–29.25]	N/A	N/A	8.5 [6.25–14.5]
**P/F ratio (mmHg)**		N/A	265.5 [331–392.25]	170 [135.75–240.5]	N/A	411.5 [386.5–436.5]	163 [129–205.75]
**Muscle Relaxant infusion**		N/A	N/A	11 (34.4%)	N/A	N/A	5 (50%)
**Acute Kidney Injury** **KDIGO** grade ≥1		0	6 (31.58%)	26 (81.25%)	0	2 (20%)	10 (100%)
**Renal Replacement Therapy**		0	0	16 (50%)	0	0	8 (80%)
**Concomitant cardiac failure**		0	0	6 (18.75%)	0	0	0
**Stress dose steroids**		0	1 (5.3%)	6 (18.75%)	0	1 (10%)	5 (50%)
**Source of Sepsis**	Respiratory	N/A	5 (26.3%)	16 (50%)	N/A	3 (30%)	4 (40%)
	Abdominal	N/A	7 (36.8%)	11 (34.4%)	N/A	2 (20%)	3 (30%)
	Skin	N/A	0	4 (12.5%)	N/A	1 (10%)	1 (10%)
	Urine	N/A	6 (31.6%)	0	N/A	4 (40%)	2 (20%)
	Osteomyelitis	N/A	1 (5.3%)	0	N/A	0	0
	Mediastinitis	N/A	0	1 (3.1%)	N/A	0	0
**Type of organism**	Gram- ve organism	N/A	16 (84%)	9 (28%)	N/A	5 (50%)	5 (50%)
	Gram+ ve organism	N/A	3 (16%)	9 (28%)	N/A	5 (50%)	2 (20%)
	Fungal	N/A	0	1 (3%)	N/A	0	0
	Empiric treatment	N/A	0	13 (41%)	N/A	0	3 (30%)
**Secondary Infections**		N/A	0	17 (53.1%)	N/A	0	5 (50%)
**Lactate on admission**		N/A	2.1 [1.15–3.57]	2.86 [2.27–4.09]	N/A	1.95 [1.6–3.1]	2.63 [1.81–3.36]
**Comorbidities ≥ 1**		19 (95%)	18 (94.7%)	26 (81.25%)	7 (70%)	9 (90%)	8 (80%)

Categorical data are presented as numbers with percentages in parentheses(). Data and parameters are presented as medians and interquartile ranges [Q1-Q3]. n, number of patients; APACHE score, Acute Physiologic Assessment and Chronic Health Evaluation; SOFA score, Sequential Organ Failure Assessment score; KDIGO Kidney Disease: Improving Global Outcomes; N/A, Not Applicable; P/F ratio, ratio of arterial oxygen partial pressure to fractional inspired oxygen.

Exclusion criteria included pre-existing immunodeficiency, immune modulating medications including steroids, chronic infection, malignancy, pre-existing liver disease, and any haematological disease.

### Phenotypic analysis of circulating leukocytes

Fresh whole blood was stained with a dead cell stain (LIVE/DEAD Fixable Aqua dead cell stain purchased from Molecular Probes, *Leiden*, *The Netherlands)* followed by fluorochrome-conjugated monoclonal antibodies (mAb) specific for cell surface expression of CD3 (clone REA613, BW264/56), CD4 (REA623), CD8 (BW135/80, REA734), CD14 (REA599, TÜK4), CD16 (REA423), CD25 (4E3), CD45RA (REA562), CD127 (REA614), CD197 (CCR7; REA546), HLA-DR (REA805), IL-12Rβ2 (REA333) and IL-23R (218213) (purchased from Miltenyi Biotec, Gladbach Bergische, Germany and R&D Systems, Abingdon, UK). Cells were stained with mAbs in PBS containing 1% bovine serum albumin and 0.02% sodium azide. Red cells were lysed with BD FACS Lysing Solution and analysed using a FACSCanto II flow cytometer (BD Biosciences) and FlowJo software (Tree Star). Lymphocytes were gated on and any doublets or dead cells were excluded from the analysis. Single stained controls were used to set compensation parameters and fluorescence-minus-one controls were used to set gates. Cell frequencies were expressed as percentages of CD3^+^ lymphocytes. Absolute numbers were determined from full blood cell counts.

dx.doi.org/10.17504/protocols.io.6zxhf7n

### Stimulation experiments

Peripheral blood mononuclear cells (PBMC) were prepared from fresh blood by density gradient centrifugation over Lymphoprep (Axis-Shield, Dundee, UK).

0.5x10^6^ cells were stimulated for 5 hours with plate-bound mAbs specific for CD3 (OKT3) and CD28 (15E8), 50 ng/ml phorbol myristate acetate (PMA) with 1 μg/ml ionomcyin, or 10 ng/ml lipopolysaccharide (LPS). Wells intended for intracellular staining for IL-23, IL-12, IL-17A and IFN-γ contained brefeldin-A.

**Fig 1:**
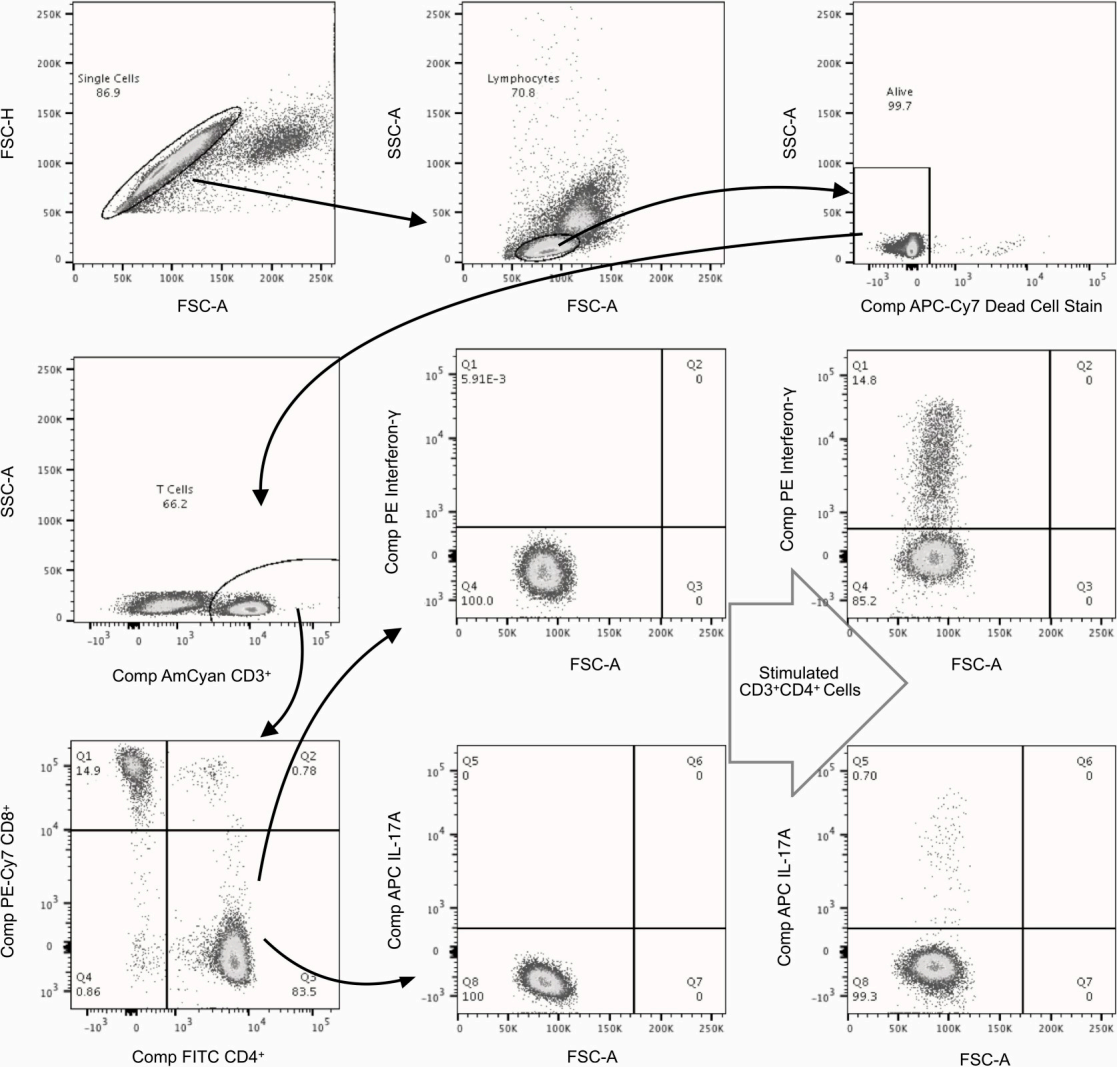
Gating strategy of flow cytometry. Gating Strategy of Flow Cytometry showing CD3^+^CD4^+^ cells expressing Interferon-γ and Interleukin17a in unstimulated and stimulated cells. Cells stimulated with phorbol myristate acetate and ionomycin.

The cells were stained with dead cell stain (LIVE/DEAD Fixable Near IR dead cell stain purchased from Thermo Fisher Scientific, Massachusetts, US) followed by fluorochrome-conjugated antibodies for labeling cell surface markers. Cells were then fixed with 4% paraformaldehyde and permeabilised with 0.2% saponin before staining with mAbs specific for intracellular IL-12 (REA121), IL-23 (727753), IL-17A (CZ8-23G1), and IFN-γ (REA600). For intracellular staining of RORγt (REA278) and T-bet (REA102) FoxP3 Staining Buffer Set was used. Once stained, the cells were fixed. The samples were acquired immediately with a BD FACS CANTO II flow cytometer and analysed using FlowJo 10.4.2 software. The gating strategy used is shown in Fig 1.

dx.doi.org/10.17504/protocols.io.6zyhf7w

dx.doi.org/10.17504/protocols.io.6zzhf76

### Statistical analysis

All statistical analysis was performed with JMP® and SPSS® Statistical Software. Differences between the three groups were analysed for continuous variables by a Wilcoxon / Kruskal-Wallis test (capped line in figures), with pair-wise comparison (n-zigzag line in figures) and Bonferoni adjusted p values. Categorical variables were compared using a Chi-Square Test. Repeated assay were analysed using a mixed effects general liner regression model, all comparisons with admission values, with Bonferroni correction for multiple comparisons. Results were considered significant for p values lower than 0.05.

## Results

The demographic characteristic of patients in this study, as outlined in Table 1, indicate that age and gender distribution was similar in all 3 groups. Patients with sepsis had greater Apache II scores (p<0.0001) and organ failure scores (p<0.0001) than patients with infection. In the phenotype study group no patients in the control or infection groups died whereas 13 (40%) of the sepsis group died.

### CD4^+^ and CD8^+^ T lymphocyte differentiation in patients with infection and sepsis

**Fig 2:**
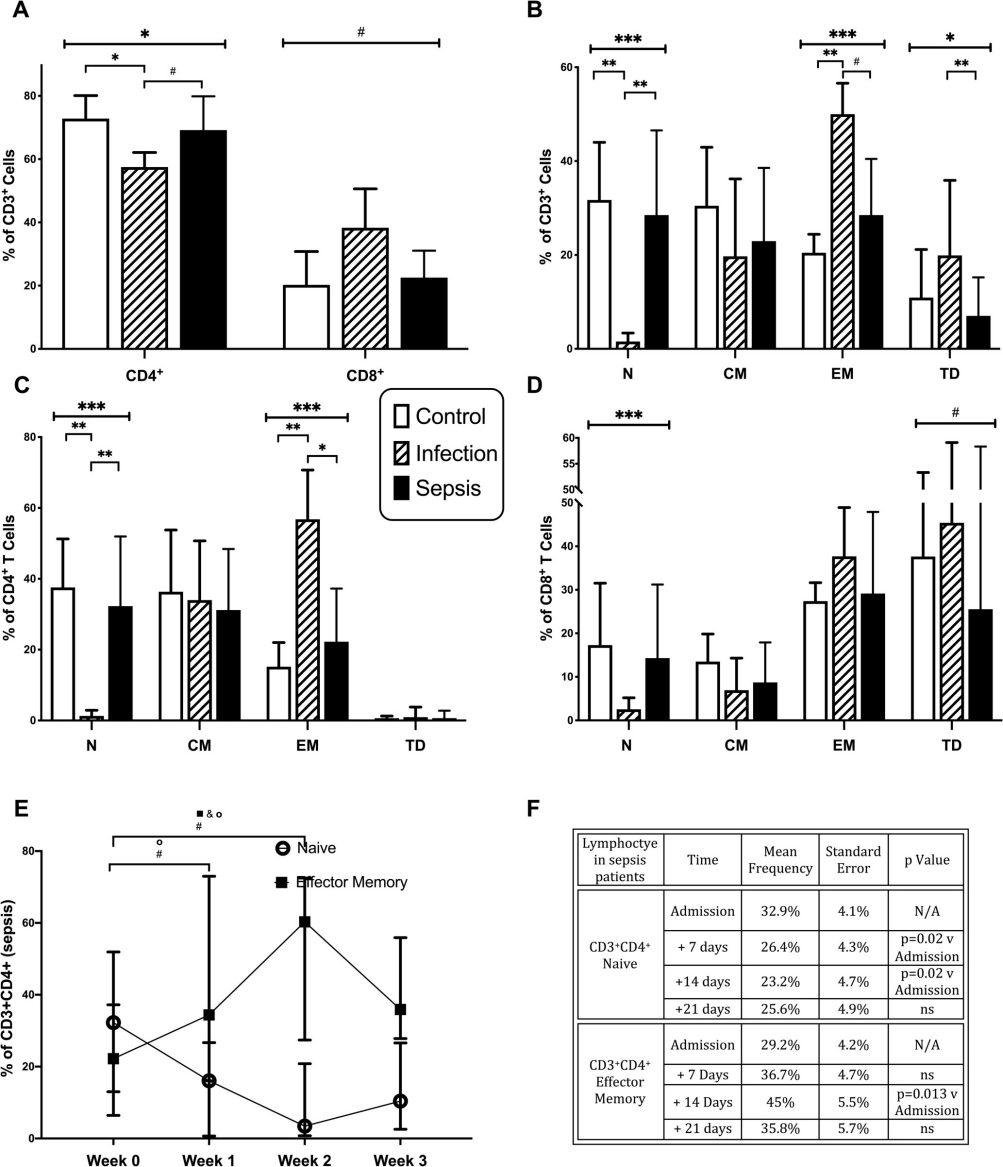
Lymphocyte differentiation by frequency. A-D, Frequencies of CD3^+^, CD4^+^, and CD8^+^ Naive (N), Central Memory (CM), Effector Memory (EM), and Terminally Differentiated (TD) T Cells in control subjects (n = 20), patients with infection (n = 19), and patients with sepsis (n = 32). Data represented as median with interquartile range. E-F, Frequencies of CD4^+^ Naïve and Effector Memory T Cells at admission (n = 32), at 7 days (n = 20), 14 days (n = 14) and 21 days (n = 15). SE = standard error of the mean. NS = non significant, N/A = Not applicable. All comparisons with admission values, with Bonferroni correction for multiple comparisons. (# = p<0.05;* = p<0.01;** = p<0.001; *** = p<0.0001).

**Fig 3:**
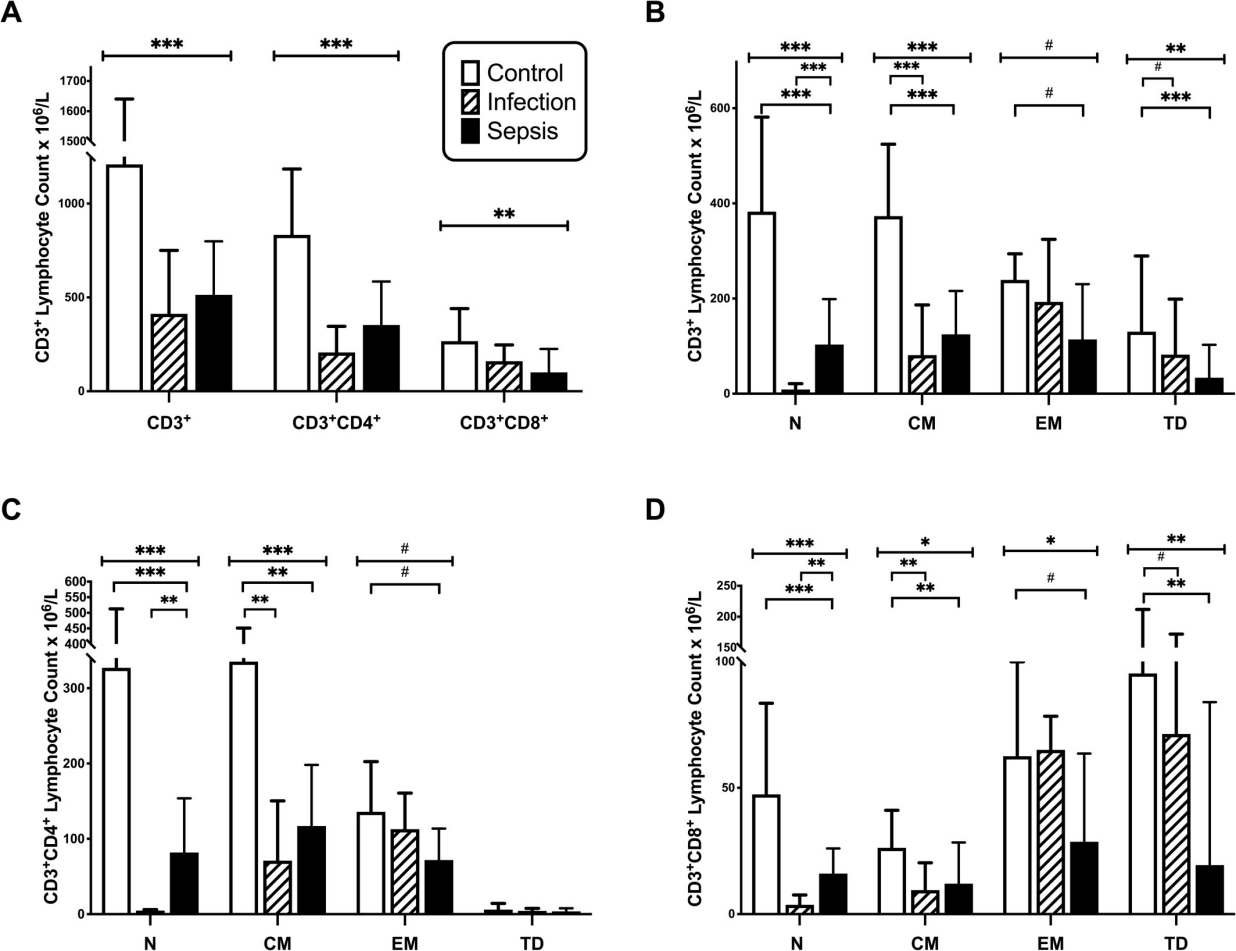
Lymphocyte differentiation by cell count. A-D. Absolute cell counts of CD3^+^, CD4^+^, and CD8^+^ Naive (N), Central Memory (CM), Effector Memory (EM), and Terminally Differentiated (TD) T Cells in control subjects (n = 20), patients with infection (n = 19), and patients with sepsis (n = 32). Data represented as median with interquartile range. (# = p<0.05;* = p<0.01;** = p<0.001; *** = p<0.0001).

The differentiation status of total CD3^+^, CD3^+^CD4^+^ and CD3^+^CD8^+^ T cells was examined by flow cytometric analysis of CD45RA and CD197 expression. The percentage frequencies of naïve (N; CD45RA^+^ CD197^+^), central memory (CM; CD45RA^-^CD197^+^), effector memory (EM; CD45RA^-^CD197^-^) and terminally differentiated (TD; CD45RA^+^CD197^-^) T cells are shown in Fig 2 and the corresponding cell counts are shown in Fig 3. The frequencies of T cells expressing CD4 were significantly lower in patients with infection without sepsis, with concomitant increases in CD8^+^ T cells (Fig 2A). Overall numbers of both CD4^+^ and CD8^+^ T cells were reduced in infection and sepsis patients (Fig 3A). The differentiation status of T cells differed across patient groups; with the frequencies of CD3^+^ naïve lymphocytes being lower in patients with infection compared to control (p<0.001) and sepsis (p<0.001) groups; and the frequencies of EM T cells being greater in patients with infection compared to control (p<0.001) and sepsis (p = 0.02) groups (Fig 2B). The percentages of CD3^+^ TD cells were greater in patients with infection than in those with sepsis (p = 0.001) (Fig 2B). The absolute numbers of naive T cells were lower in patients with sepsis than in controls (p<0.0001) and lower in patients with infection than in septic patients (p<0.0001) (Fig 3B). CD3^+^ CM counts were lower in patients with infection (p<0.0001) and with sepsis (p<0.0001) compared to controls. CD3^+^ EM counts were lower in septic patients (p = 0.014) than in controls, while CD3^+^ TD counts were lower in patients with infection (p = 0.025) and sepsis (p<0.0001) compared to controls.

The percentages of CD4^+^ T cells with naïve phenotypes was lower in patients with infection compared to the control (p<0.001) and sepsis (p<0.001) groups; whereas percentages of EM CD4^+^ T cells was greater in patients with infection than in the control (p<0.001) and sepsis (p = 0.007) groups (Fig 2C). A similar pattern was observed for CD8^+^ lymphocyte differentiation, with the exception that higher frequencies of CD8^+^ cells from all subject groups expressed TD phenotypes. (Fig 2D).

Absolute numbers of CD4^+^ T cells were lower in patients with infection and sepsis compared to controls (p<0.001) (Fig 3C). Within the CD4^+^ T cell compartment, naïve cell counts were lower in sepsis patients than in controls (p = 0.0001) and lower with patients with infection than in those with sepsis (p<0.001). CM CD4^+^ T cell counts were lower in patients with sepsis than in healthy controls (p<0.001) and lower in patients with infection than healthy controls (p<0.001). EM CD4^+^ T cell counts were lower in patients with sepsis than healthy controls (p = 0.011). CD4^+^ TD lymphocyte counts were similarly low in all three groups (Fig 3C).

Similar results were observed with CD8^+^ T cell counts, except that TD cells were more prominent among CD8^+^ than CD4^+^ T lymphocytes, and were lower in patients with infection (p = 0.018) and sepsis (p = 0.001) compared to controls (Fig 3D).

Thus, at the start of illness, both CD4^+^ and CD8^+^ T lymphocytes were activated in patients with infection, but not patients with sepsis. However, in the sepsis group, the percentage of CD3^+^CD4^+^ naive cells decreased over time (p = 0.025) (Fig 2E). CD4^+^ naive cells decreased in the two weeks after the onset of sepsis (Fig 2F). The percentage of CD3^+^CD4^+^ effector memory cells increased over time (p = 0.02) (Fig 2E and 2F) with CD4^+^ effector memory cells increasing two weeks after the onset of sepsis (Fig 2E and 2F). There was no change in CD8^+^ activation over the same time.

### IL-23 production by monocytes is impaired in patients with sepsis

**Fig 4:**
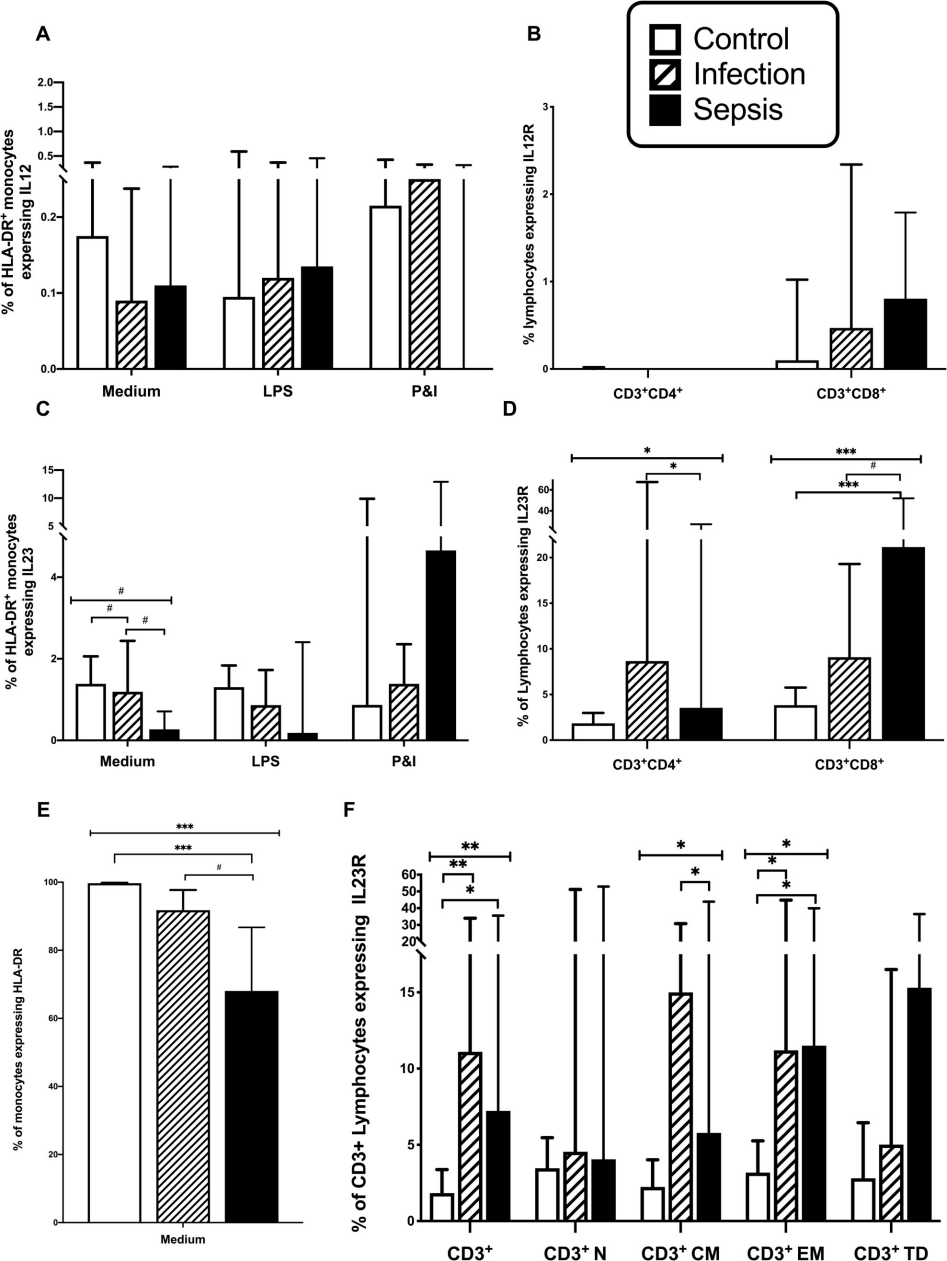
Monocyte expression of HLA-DR, IL12, and IL23 and lymphocyte expression of IL12R and IL23R. A; HLA-DR^+^ monocytes expressing IL12. B; T Lymphocytes expressing IL12R. C; HLA-DR^+^ monocytes expressing IL23. D; T Lymphocytes expressing IL23R. E; Monocytes expressing HLA-DR^+^. F; T lymphocytes expressing IL23R by differentiation status, ie Naive (N), Central Memory (CM), Effector Memory (EM), and Terminally Differentiated (TD). Control subjects (n = 20 in T cell group, 10 in monocyte group), patients with infection (n = 19 in T cell group, 10 in monocyte group), and patients with sepsis (n = 32 in T cell group, 10 in monocyte group). Monocytes are unstimulated (medium) or stimulated with lipopolysaccharide (LPS) or phorbol myristate acetate (PMA) and ionomycin. Data represented as median with interquartile range. (# = p<0.05;* = p<0.01;** = p<0.001; *** = p<0.0001).

As previously reported, lower frequencies of CD14^+^ monocytes expressed HLA-DR in patients with sepsis compared to control subjects (p<0.0001) (Fig 4E), and patients with infections only (p = 0.038), potentially accounting for the observed failure of lymphocyte activation in patients with sepsis. Few CD14^+^ monocytes expressed either IL-12 or IL-23 upon stimulation with LPS or PMA and ionomycin (Fig 4A and 4C). Unstimulated monocytes from septic patients exhibited lower expression of IL-23 than monocytes from control subjects (p = 0.038) and patient’s with infection (p = 0.021) (Fig 4C), suggesting that Th17 cell differentiation may be impaired in septic patients.

### Transcription factors in CD3^+^ cells

**Fig 5:**
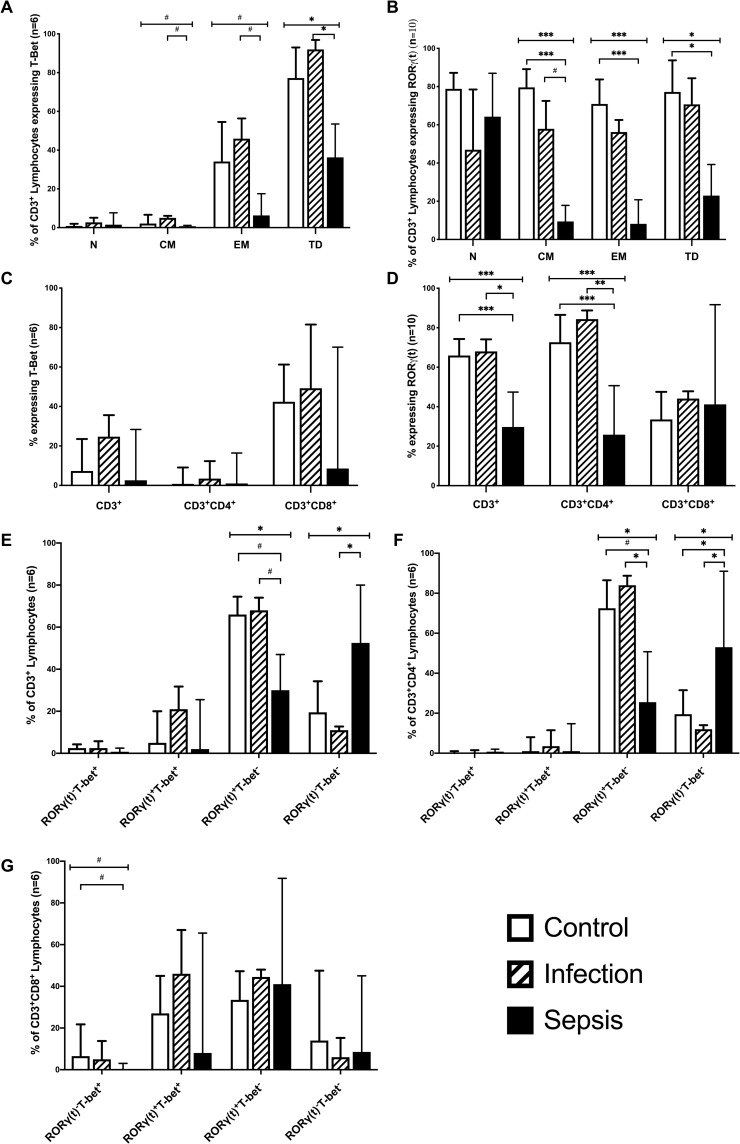
Frequency of RORγt and T-bet expression in CD3^+^, CD3^+^CD4^+^, CD3^+^CD8^+^ lymphocytes. A-G. Expression of transcription factors RORγt and T-bet in CD3^+^, CD3^+^CD4^+^, and CD3^+^CD8^+^ Lymphocytes and in CD3+ Naive (N), Central Memory (CM), Effector Memory (EM), and Terminally Differentiated (TD) Cells. Co-expression of T-bet in CD3+, CD3^+^CD4^+^, and CD3^+^CD8^+^ lymphocytes was also analysed in 4E-G where RORγ(t)^-^T-bet^+^ only expressed T-bet^+^; RORγ(t)^+^T-bet^+^ co-expressed the two transcription factors; RORγ(t)^+^T-Bet^-^ only expresed RORγ(t)^+^; and RORγ(t)^-^T-Bet^-^ denoted cells that expressed neither. n, number of patients. Data represented as median with interquartile range. (# = p<0.05;* = p<0.01;** = p<0.001; *** = p<0.0001).

We next investigated the expression of the Th1-associated transcription factor T-bet and the Th17-associated transcription factor RORγt by total T Cells, CD4^+^, and CD8^+^ T cells from the three subject groups. RORγt was found to be expressed by lower frequencies of T cells from patients with sepsis compared to patients with infections only (p = 0.003) and control subjects (p<0.0001) (Fig 5D). RORγt expression by naïve T cells was similar in all 3 patient groups, but it was significantly lower in CM and EM T cells from septic patients compared to controls (p<0.0001) and lower in CM from sepsis compared to infected patients (p = 0.034). (Fig 5B) Thus sepsis was associated with reduced expression of the Th17 transcription factor RORγt in effector, central memory, and terminally differentiated CD3^+^ lymphocytes.

There was no significant difference in the expression of T-bet by total T cells, CD4+ and CD8+ T cells (Fig 5C). T-bet expression was similar in naïve T cells in all groups, but it was lower in CM (p = 0.018), EM (p = 0.019), and TD (p = 0.003) T cells from sepsis patients compared to patients with infections only. Collectively these data suggests that sepsis is associated with a failure of T cell activation to both Th1 and Th17 phenotypes. An underlying lymphocyte anomaly seems unlikely in septic patients as T-bet and RORγt transcription factor expression was similar in Naïve T cells from all three subject groups. The predominance of RORγt expression in CD3^+^ lymphocytes with sepsis highlights the importance of the Th-17 response in sepsis.

### Transcription factors in CD4^+^ and CD8^+^ cells

Most CD4^+^ T cells from control subjects and patients with infections only expressed RORγt, with very few CD4^+^ T cells expressing T-bet (Fig 5C, 5D and 5F). The expression of RORγt by CD4^+^ T cells was significantly lower in septic patients than in infected patients (p = 0.001) or healthy controls (p = 0.0001).

CD8^+^ T cells from control subjects and patients with infections only frequently expressed both T-bet and RORγt, (Fig 5C and 5D). In patients with sepsis compared to control the expression of T-bet was significantly lower (p = 0.02) in CD3^+^CD8^+^ cells that did not co-express RORγt. Co-expression of RORγt and T-bet was simliar in all three groups (Fig 5G).

The decreased expression of the Th17 signature transcription factor in effector CD4^+^ T lymphocytes of patients with sepsis, but normal RORγt expression in naïve CD4^+^ T cells, suggests a failure of CD4^+^ Th17 differentiation in these patients. Similarly sepsis appears to be associated with a failure of CD8^+^ Th1 cell differentiation, rather than an underlying defect in lymphocyte functionality.

### IL-12R and IL-23R expression by T cells from patients with sepsis

The Th-1 lymphocyte phenotype is characterised by surface expression of IL-12R, while surface expression of IL-23R characterises Th17 lymphocytes. Few CD3^+^ lymphocytes from healthy volunteers expressed either IL-12R or IL-23R (Fig 4B and 4D), which is consistent with existing literature in patients [19, 20]. However IL23R expression was more frequent in patients with infection (p = 0.001) and sepsis (p = 0.002), compared with healthy controls (Fig 4F). IL-23R expression by naïve and TD T cells was similar in the three patient groups. However IL-23R expression was greater in CM T cells from patients with infection compared with sepsis (p = 0.01) and was greater in EM T cells from patients with sepsis (p = 0.008) and from patients with infection (p = 0.003) compared with healthy controls. This data points to a Th17 immune response taking place in patients with infection which is attenuated in septic patients.

In CD4^+^ T cells, IL-23R expression was greater in patients with infection than sepsis (p = 0.005), while in CD8^+^ T cells, IL-23R expression was greater in patients with sepsis than with patients with infection (p = 0.04) and controls (p<0.001) (Fig 4D). Expression of the IL-12R was rare in CD4^+^ and CD8^+^ T cells and was similar in all patient groups (Fig 4B).

As expression of the IL-23R is a hallmark of Th17 phenotype, our data suggest that immunity to infection is predominantly based upon a Th17 response, rather than a Th1 response. Furthermore as IL-23R expression was greater in CD3^+^ and CD3^+^CD4^+^ lymphocytes of patients with infection, who did not have multiple organ failure, then an exaggerated Th17 response in circulating lymphocytes does not appear to be the basis of sepsis induced organ failure. Lastly the Th17 response was preserved in CD8^+^ lymphocytes. As only 10% of CD4^+^RORγt^+^ T cells from patients with infection expressed IL-23R, this suggests that activation of a relatively small proportion of the potential pool of CD4^+^ Th17 cells is required to control infection.

### Interferon-γ and IL-17A production by stimulated T cells from patient and controls

**Fig 6:**
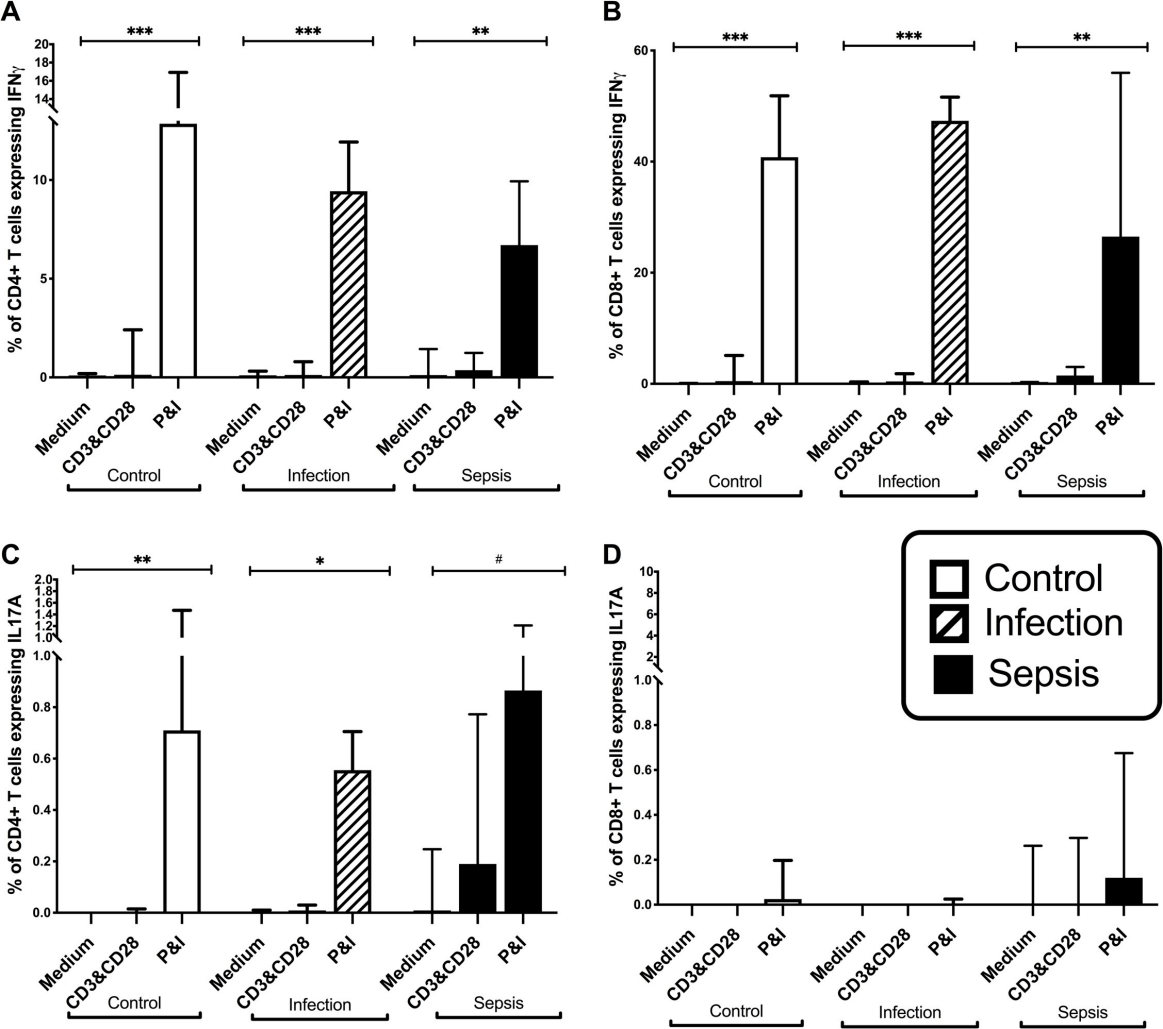
Frequency of IFNγ and IL17A expression on stimulated CD3^+^CD4^+^and CD3^+^CD8^+^ lymphocytes. A-D, Frequencies of Interferon-γ (IFNγ) and IL17A expressing CD4^+^ and CD8^+^ T Cells in control subjects (n = 20), patients with infection (n = 19), and patients with sepsis (n = 32). Medium are unstimulated cells, CD3&CD28 is stimulation with CD3 and CD28 antibodies, P&I is stimulation with Phorbol Myristate Acetate and Ionomcyin. Data represented as median with interquartile range. (#p<0.05;*p<0.01;**p<0.001; ***p<0.0001).

CD4^+^ and CD8^+^ T cells from patients and healthy controls expressed little or no IFN-γor IL-17A without *in vitro* stimulation (Fig 6A–6D), which is consistent with existing literature [21]. However stimulation with PMA and ionomycin, induced IFN-γ production by significant numbers of CD4^+^ and CD8^+^ T cells and IL-17A by smaller numbers of CD4^+^ T cells (Fig 6A–6D). The frequencies of CD4^+^ and CD8^+^ T cells that produced IFN-γ or IL-17 in response to any mode of stimulation were similar using PBMC from healthy donors and patients with infection only or sepsis. These data suggest that CD4^+^ and CD8^+^ T cells from patients with sepsis are not defective in their ability to produce IFN-γ or IL-17, but may be failing to receive stimulatory and/or Th1/Th17 polarisation signals from cells of the innate immune system.

The frequencies of Treg lymphocytes, defined by the CD3^+^CD4^+^CD25^+^CD127^−^ phenotype, were significantly different across patient groups, (p = 0.008), being greatest in patients with sepsis, (median 11.7%, IQR 7.5–14.6%), than with infection (Median 7.8%, IQR 6–10%) and healthy controls (Median 7.7%, IQR 6.7–9.2%).

## Discussion

In this study the clinical presentation of infection was linked with specific attributes of host immunity; in septic patients there was a complex failure to differentiate T lymphocytes to an activated phenotype at onset of illness, in particular CD8^+^ Th1 cells and CD4^+^ Th17 cells, while simultaneously expanding an inhibitory population of Treg cells. While septic patients eventually activated circulating T lymphocytes, this was only after a considerable delay.

In our study of human sepsis, expression of the signature Th17 transcription factor and of surface IL-23R were reduced in CD4^+^ lymphocytes of septic patients. Indeed patients who tolerated infection with relative impunity, without developing organ failure, elaborated a more robust Th17 response, which may potentially be protective in this context. Accordingly the Th17 lymphocyte response, may not account for systemic inflammation and multiple organ failure, as suggested in prior animal studies of sepsis [22]. The absence of IL-12 receptor expression in both CD4^+^ and CD8^+^ lymphocytes, which is consistent with existing human data suggests that immunity in elderly humans with sepsis is predominantly mediated by a Th-17 rather than a Th-1 response [20].

In human sepsis the relative importance of the Th17 response in driving inflammation and or providing immunity is unclear. The IL-23/IL-17 axis is involved in systemic inflammation in murine models of infection[22]. However in humans, and in line with our findings, Ronit *et al* reported a decrease in Th17 lymphocytes, with endotoxin induced systemic inflammation [23]. In contrast Mikacenic *et al*. reported elevated IL-17 in the broncho-alveolar lavage fluid of patients with acute respiratory distress syndrome (ARDS), with IL-17 levels correlating with the severity of multiple organ failure [24]. Similarly Zan *et al*., also studying patients with sepsis induced ARDS, reported elevated IL-17 in sepsis non-survivors [25]. Furthermore, Brunialti *et al*, reported that T lymphocytes expressing IL-17 were increased in patients with sepsis [26], and Maravista *et al*, reported IL-17 as the only cytokine produced in excess by shocked septic patients, [27]. In a separate and distinct context, a study of neonatal sepsis reported that mortality was linked with excess levels of IL-17[28]. However not all studies have found an association between IL-17 and outcome in sepsis [29] [30].

Crucially none of the aforementioned studies included patients with infection who did not develop sepsis, and thus did not adequately explore the link between Th17 responses and sepsis outcomes. The present study, by including a cohort of patients with infection without sepsis, indicates that the Th-17 response may have an essential role in mediating protective immune responses in humans with infection.

This concept that human sepsis is fundamentally related to a failure to elaborate a robust adaptive immune response is consistent with the current appreciation of the significance of immune suppression in patients with sepsis [31]. This study suggests a specific failure of the CD4^+^ effector memory lymphocyte mediated Th-17 response. This is plausible as IL-17 enhances polymorphonuclear chemotaxis and activation, and is crucially important in mediating mucosal immunity, specifically for systemic *Candida* infections, which account for 20% of all infections in critically ill patients [32–34]. Furthermore IL-23 responsiveness of human CD8^+^ memory lymphocytes decreases with age [35], and there is lower potential for inducible CD4^+^/IL-23r with age [36] and this decrease in Th-17 responsiveness with age could potentially contribute to the adverse sepsis outcomes observed in the elderly.

As in other studies, HLA-DR expression by monocytes from septic patients was decreased [8]; which likely accounted for the observed failure of T lymphocyte activation. However, in T cell activation, there is an additive effect of co-stimulatory cytokines from the IL-12 family [37]. IL-12 drives CD4^+^ T cell differentiation into IFN-γ-secreting Th1 cells, whereas IL-23 in concert with IL-1β, IL-6 and TGFβ-1 drives activated CD4^+^ differentiation to a Th17/IL-17 secreting phenotype [38] [39]. In this study, IL-23 production by CD14^+^ monocytes from septic patients was decreased which would inhibit a robust Th-17 response [37].

## Limitations

This study was not designed or powered to detect an association between specific markers of a Th17 adaptive immune response and either the occurrence of nosocomial infection, or the incidence of mortality in critically ill patients. A larger study will be required to test these associations and to test the utility of specific indices of T lymphocyte activation as clinical biomarkers of the risk for nosocomial infection and for sepsis mortality.

This analysis of circulating T lymphocyte activation and phenotype may not reflect the status of tissue bound lymphocytes. While including a group of patients with infection in the study design provides evidence of circulating T lymphocyte activation in patients with infection who do not develop sepsis, this still leaves questions regarding the activation of tissue bound lymphocytes largely unanswered. The results of this study suggest a link between T lymphocyte activation and the occurrence of sepsis in patients with infection, but does not differentiate between a causal and coincidental link. The observation of T lymphocyte activation in patients with infection provides at best inferential evidence of causal immune mediation of patients’ response to infection. Lastly the study was designed to recruit patients with an infection or who had established sepsis and does provide insight to the earlier stages of illness.

However the results of this study clearly link delayed activation of CD4^+^Th17 lymphocytes with sepsis in elderly humans and provide biomarkers for the occurrence of sepsis in the elderly that have the potential to inform subsequent studies of immune modulation in elderly patients with sepsis.
